# Non-contact profilometry of eroded and abraded enamel irradiated with an Er:YAG laser

**DOI:** 10.1590/1678-7757-2017-0029

**Published:** 2018-04-18

**Authors:** Renata Siqueira Scatolin, Vivian Colucci, Taísa Penazzo Lepri, Adílis Kalina de Alexandria, Lucianne Cople Maia, Rodrigo Galo, Maria Cristina Borsatto, Silmara Aparecida Milori Corona

**Affiliations:** 1 Universidade de São Paulo, Faculdade de Odontologia de Ribeirão Preto, Departamento de Odontologia Restauradora, Ribeirão Preto, São Paulo, Brasil; 2 Universidade de Ribeirão Preto, Departamento de Odontologia, Ribeirão Preto, São Paulo, Brasil; 3 Universidade Federal do Rio de Janeiro, Faculdade de Odontologia, Departamento de Odontopediatria e Ortodontia, Rio de Janeiro, Rio de Janeiro, Brasil; 4 Universidade do Vale do Jequitinhonha e Mucuri, Departamento de Odontologia, Diamantina, Minas Gerais, Brasil; 5 Universidade de São Paulo, Faculdade de Odontologia de Ribeirão Preto, Departamento de Clínica Infantil, Ribeirão Preto, São Paulo, Brasil

**Keywords:** Citric acid, Enamel, Laser, Tooth abrasion, Tooth erosion

## Abstract

Literature has reported positive results regarding the use of lasers in the control of erosive lesions; however, evaluating whether they are effective in the control of the progression of erosive/abrasive lesions is important.

**Objectives:**

This study aimed to evaluate the effect of the Er:YAG laser irradiation in controlling the progression of erosion associated with abrasive lesions in enamel.

**Material and methods:**

Bovine incisors were sectioned, flattened and polished. Forty-eight enamel slabs were subjected to treatment in an intraoral phase. Twelve volunteers used an intraoral appliance containing one slab that was irradiated with an Er:YAG laser (5.2 J/cm^2^, 85 mJ, 2 Hz) and another non-irradiated slab on each side of the appliance, during one phase of 5 d, under a split-mouth design. Devices were subjected to erosive challenges (1% citric acid, 5 min, 3 times a day) and abrasive challenges one h after (brushing force of 1.5 N for 15 s) randomly and independently on each side of the device. Measurements of enamel loss were performed via 3D optical profilometry (μm). We analyzed data using the Kruskal-Wallis and Mann-Whitney tests and morphological characteristics via scanning electron microscopy.

**Results:**

Following erosive and abrasive challenges, the group that was irradiated with the Er:YAG laser presented less loss of structure than the non-irradiated group. The group that underwent erosion and irradiation did not exhibit a significant difference from the non-irradiated group.

**Conclusion:**

Irradiation with the Er:YAG laser did not control the loss of structure of enamel subjected to erosion but did control abrasion after erosion.

## Introduction

Dental erosion occurs through the action of intrinsic or extrinsic acids and without the involvement of bacteria^23^. The demineralization caused by erosion is initially characterized by a softening of the surface and is followed by continuous dissolution of enamel crystals, which leads to the loss of hard dental tissue^13^. In this weakened state, dental surfaces are more prone to wear via abrasive action^19^. The most common form of abrasion is brushing, and factors such as the brushing technique and force, the stiffness of the toothbrush bristles and the abrasiveness of the toothpaste used may be involved in this process^16^.

Tooth structure loss due to erosive and abrasive challenges is irreversible, and several strategies have been developed with the aim of preventing damage, including the use of high-intensity lasers^12^. To increase the acid resistance of the enamel, the use of an Er:YAG laser has been proposed^4^ ^,^ ^7^.

Irradiation with an Er:YAG laser can promote partial denaturation of the enamel matrix, forming a mineral block that makes diffusion of acids within the tissue difficult^28^. Additionally, it might prevent the progression of erosive lesions and therefore minimize the wear caused by abrasion. Another hypothesis regarding the mechanism by which the laser increases the acid resistance of the enamel is that the irradiation temperature between 100 and 650°C can reduce the amount of water and carbonate in the tissue, resulting in increased resistance against acid^8^. Some studies have even suggested that the increasing acid resistance of the enamel is related to morphological changes in the tissue^10^ ^,^ ^11^. When the enamel and dentin are irradiated with a laser, the surfaces are partially melted and solidified^10^ ^,^ ^11^. which suggests that the enamel surface would be less permeable.

Because of the susceptibility of enamel to the development of erosive and abrasive lesions, the search for methods capable of controlling such lesions has been intensified. Although the use of lasers to control erosive lesions is widely presented in the literature, there are no studies assessing the use of an Er:YAG laser to control the progression of erosive/abrasive lesions in enamel.

The null hypothesis tested was that the losses of enamel structure following erosive challenges versus erosive challenges associated with abrasive challenges are similar in slabs treated or untreated with an Er:YAG laser.

## Material and methods

### Experimental design

This *in situ,* split-mouth, double-blind study with one phase of 5 d was approved by the Ethics Committee of the School of Dentistry of Ribeirão Preto (process number: 2010.1.552.58.7). Forty-eight sound enamel slabs were subjected to the initial erosive challenge, after which they were randomly assigned to 4 groups (n=12). The factors under study were the type of wear at 2 levels (a. erosion and b. erosion associated with abrasion) and Er:YAG laser irradiation at 2 levels (I. irradiated and II. non-irradiated). After treatment, fragments were exposed to erosive wear on one side of the device and erosive wear associated with abrasive wear on the other side of the device during the *in situ* phase. The response variable was obtained based on enamel loss evaluated with a 3D optical profilometer. We analyzed morphological characteristics of surface via scanning electron microscopy.

### Preparation of enamel slabs

Bovine incisors were freshly extracted and stored in a 0.1% thymol solution at 4°C and were then examined with a stereomicroscope (Leica S6 D Stereozoom, Mycrosystems Leica AG, Heerbrugg, Switzerland) at a magnification of 40x. Teeth with structural anomalies or cracks were discarded^22^. Dental crowns were sectioned with a diamond disk (15HC, Buehler, Lake Bluff, IL, USA) using a sectioning machine (Isomet 1000; Buehler, Lake Bluff, IL, USA), resulting in two enamel slabs *per* tooth (5×3x2.5 mm). Enamel surfaces of these slabs were flattened in a water-cooled polishing machine for 20 s (Phonix β, Buehler, Lake Bluff, IL, USA) using Al_2_O_3_ papers (#600 and #1200; Norton Abrasivos Ltda; Guarulhos, SP, Brazil) with a standardized strength of 20 N^22^. Slabs were polished using a 0.3-μm alumina suspension (Buehler, Lake Bluff, IL, USA), and a standardized strength of 20 N for 60 s was applied. This procedure was performed with a device in which the specimens were fixed, and a standardization of the time and strength of the procedures was achieved. Fragments were sterilized by microwave irradiation (650 W/3 min)^24^, and those that exhibited cracks were excluded.

Three microhardness measurements were performed in the center of the specimen, with a 100-μm distance between each measurement, using a HMV-2000 microhardness tester (Shimadzu Corporation, Tokyo, Honshu, Japan). A diamond indenter was used to test the Knoop hardness (KHN), and a static load of 25 g for 5 s was applied^14^. The 48 selected slabs, averaging 330 KHN (±10%), were divided into for groups.

Specimens were waterproofed with three layers of acid-resistant nail varnish (Colorama, São Paulo, SP, Brazil) while maintaining the vestibular surface, on which the specimen was delimited into the following 4 distinct areas: 1- sound (reference area); 2- initial erosion; 3- treatment (irradiated or non-irradiated with the Er:YAG laser), and 4- after the *in situ* phase.

**Figure 1:**
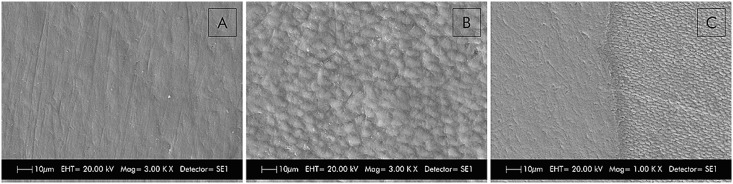
Representative images of scanning electronic microscopy. A- Sound enamel; B- Eroded enamel; C- Image shown the erosive effect only on the surface that did not receive the composite resin as insulation material

To create a reference area, a layer of composite Filtek Z250 resin (3M/ESPE, Saint Paul, MN, USA) was inserted without acid etching or an adhesive system in the first delimited area. This procedure allowed each slab to retain a sound reference area that did not undergo any treatment or erosive or erosive+abrasive challenge. Scanning electronic microscopy imaging (Figure 1A, B and C) was performed to verify that the use of the composite resin as insulation material would not interfere with the topography of the enamel surface and would prevent the penetration of citric acid into the isolated surface.

### Initial erosive challenge

To simulate previous erosive lesions, specimens were subjected to two erosive challenges in 20 ml of 1% citric acid (pH 2.3) for 5 min, twice a day, for 2 d^22^. These challenges were conducted in an orbital shaker (CT155, Cientec, Piracicaba, SP, Brazil) with a stirring rate of 100 rpm. Following the initial erosive challenge, specimens were rinsed for 10 s with deionized water and stored in 10 ml of artificial saliva (pH = 7) at 37°C between challenges and at night. The artificial saliva was similar to that described by McKnight-Hanes and Whitford^17^ (1992) and modified by Amaechi, et al.^1^ (1999). It was composed of methyl-p-hydroxybenzoate (2.0 g), sodium carboxymethylcellulose (10.0 g), KCl (0.625 g), MgCl_2_.6H_2_O (0.059 g), CaCl_2_.2H_2_O (0.166 g), K_2_HPO_4_ (0.804 g), and KH_2_PO_4_ (0.326 g) in 1000 ml of water solution.

Following the initial formation of erosion-like lesions, a new part of the specimen was also covered with the resin composite without etching or the application of an adhesive system.

### Surface treatment

Twenty-four slabs received Er:YAG laser irradiation on the enamel surface (Fidelis Er III, Fotona, Ljubljana, Slovenia), and 24 slabs did not receive treatment. Irradiation was performed in non-contact mode (handpiece no. R02-C-1122), unfocused, at a distance of 25 mm from the specimen. We applied the following parameters: 5.2 J/cm^2^ energy density, 85 mJ, frequency of 2 Hz, and spot diameter of 0.9 mm, under water spray (3.0 ml/min). Each specimen was irradiated for approximately 10 s. Non-irradiated specimens were kept in relative humidity until the beginning of the *in situ* phase. After performing surface treatments, a protective layer of composite resin was placed on the specimen to cover the other part of the surface (irradiated or non-irradiated).

### Selection of volunteers and the intraoral phase

We selected volunteers (n=12) of both genders with a mean age of 26 years who presented a normal salivary flow, an absence of active caries lesions, and a salivary buffer with a pH between 6.5 and 7.0 and who had the availability to follow the schedule established. Volunteers with systemic diseases and digestive disorders and those who were pregnant, smokers or on medication that could interfere with salivary secretion were excluded from the study.

Each volunteer had an impression of his/her maxillary arch recorded to produce an intraoral appliance that was constructed in acrylic resin. Four fragments were fixed with two slabs on each side, one of which was irradiated, while the other was non-irradiated, during one phase of 5 d, under a split-mouth design.

Fragments were fixed with wax 1 mm below the edge of the palatal appliance to prevent abrasion by contact with the surface of the tongue.

### Erosive challenges and erosive challenges associated with abrasive challenges

For the 2-day lead-in period, the volunteers were instructed to brush their teeth exclusively with the toothbrush (Oral-B Indicator 35, Gillette do Brazil Ltda., Manaus, AM, Brazil) and dentifrice (Colgate Maximum Protection Caries, Colgate-Palmolive, Osasco, SP, Brazil) provided by the researchers. After this period, the erosive challenges or erosive challenges associated with abrasive challenges on the enamel surface began. The erosive challenge was performed 3 times *per* day (8, 12 and 16 h) to simulate the contact of the volunteer with acidic drinks and foods 3 times a day. Each challenge consisted of removing the palatal device from the oral cavity and immersing it in 100 ml of 1% citric acid (pH 2.3) for 5 min^22^. We needed 100 mL to cover the palatal appliance, which was subsequently rinsed for 20 s under running water and reinserted into the mouth.

**Figure 2:**
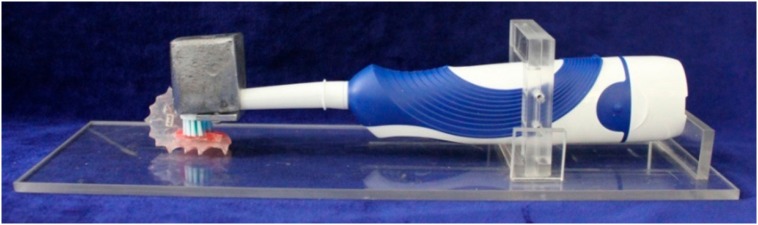
Illustrative image of the device used for brushing procedures

The abrasive wear was generated randomly for each volunteer. It was performed on one side of the palatal device (left or right) one hour after the erosive challenges. Specimens were brushed *ex vivo* with the aid of electric toothbrushes with soft bristles and rounded tips (Oral B Pro Health, Gillette do Brazil Ltda., Manaus, AM, Brazil) using a fluoridated toothpaste slurry (3 g of toothpaste/10 ml of water; Colgate Maximum Protection Caries, Colgate-Palmolive, Osasco, SP, Brazil). The electronic toothbrush was attached to an apparatus during the brushing movements (Figure 2) that allowed a brushing force of 1.5 N^21^ to be applied for 15 s (166 oscillations)^15^ for each specimen. We performed this procedure three times each day (9, 13 and 17 h). Volunteers were individually trained and instructed to perform this procedure. These challenges began on the second day of the use of the device to allow the formation of the acquired pellicle.

Biofilm control was performed at the end of each experimental day by dripping 0.2% chlorhexidine (Bioquanti Manipulation, Ribeirão Preto, SP, Brazil) on the fragments for 1 min followed by rinsing with tap water.

### Three-dimensional optical profilometry analysis

After the end of the *in situ* phase, all of the composite resin was carefully removed to avoid compromising the adjacent enamel using a type 11 scalpel blade positioned at the tooth/resin interface^22^. Thus, specimens had four distinct areas: sound, initial erosion, treatment (irradiated or non-irradiated), and after the *in situ* phase.

Resultant topographical changes were determined with the aid of a 3D non-contact optical profilometer (PS50 Optical Profilometer, Nanovea^®^, Irvine, CA, USA) that provides high accuracy regardless of study object, roughness level, flatness, illumination and measurement speed. This device allowed for the scanning of an area of 1.5 mm in length (x-axis) by 4 mm wide (y-axis). To guarantee the same level of flattening of all specimens, a parallelometer was used before the 3D non-contact profilometry.

We captured measurements with a chromatic confocal sensor using a white light axial source, a scan velocity of 2 mm/s and a refraction index of 10,000. The average structural loss corresponded to the size (in μm) of the gap between the experimental areas (initial erosion, treatment and final *in situ* phase) and the control area (sound), which we measured using a resource from the Nanovea Professional 3D program. Three linear measurements were performed, involving the following areas: 1) the sound area compared with the initial erosion area, 2) the sound area compared with the treatment area, and 3) the sound area compared with the final *in situ* phase area. We conducted all measurements in triplicate, and then calculated the mean values.

### Scanning electron microscopy analysis

After profilometry readings, we randomly selected three specimens of each group for SEM analysis. Specimens were cleaned by ultrasound (Ultrasonic Cleaner T-1449-D, Odontobrás, Ribeirão Preto, SP, Brazil) for 10 min to remove any residues and then immersed into a glutaraldehyde solution (2.5%) in sodium cacodylate (0.1 M) buffer with a pH of 7.4 (Merck KGaA, Darmstadt, Hessen, Germany). Samples were dehydrated in an increasing series of 20, 50, 75, 95, and 100% ethanol (Labsynth Ltda., Diadema, SP, Brazil) for 20, 20, 20, 30, and 60 min, respectively. Specimens were metalized with a fine gold overlay (Bal- Tec, SCD 050 Sputter Coater, Balzers, Liechtenstein), submitted to SEM (Zeiss, EVO 50, Cambridge, England) and photographed at a magnification of 3000x so that the surfaces could be analyzed^22^.

### Statistical analysis

We performed sample size calculation considering a maximum error of 5%, obtaining a sample size of 10. With an addition to the sample size of 20% considering sample loss, we established n = 12 for this study.

We performed analysis of the data obtained through profilometry using SPSS 12.0 (SPSS Inc., Chicago, IL, USA) for Windows with a significance level of 5%. A normality test (Kolmogorov-Smirnov) was performed to check data normality. Because the distribution was not normal, we calculated the mean values, and analyzed the data using the Kruskal-Wallis test with the following factors being used for comparison: laser irradiation (irradiated or non-irradiated) and the type of challenge (erosion or erosion associated with abrasion). We carried out multiple comparisons using the Mann-Whitney test.

## Results

### Profilometry analysis

Data analysis revealed statistically significant differences between the groups after the *in situ* phase. The results are shown in Table 1.

**Table 1:** Mean (SD) the enamel structure loss μm) measured by the gap between the experimental conditions and control area

	Non-irradiated/ Eroded in intraoral phase	Non-irradiated/Eroded and abrasioned in intraoral phase	Irradiated/Eroded in intraoral phase	Irradiated/Eroded and abrasioned in intraoral phase
Sound area - Initial erosion area	13.71(3.40)^aA^	14.50(3.81)^aA^	14.31(4.03)^aA^	11.98(2.72)^aA^
Sound area - Treatment area	13.49(3.75)^aA^	15.48(3.66)^aA^	14.80(5.03)^aA^	12.84(4.13)^aA^
Sound area - Final *in situ* phase area	31.69(10.68)^bA^	37.36(10.60)^bB^	34.22(11.04)^bA^	33.10(9.20)^bA^

* Lower case letters - indicating statistical analysis between rows

** Capital letters - indicating statistical analysis between columns

Following the erosive challenges associated with the abrasive challenges, the group that was irradiated with the Er:YAG laser had significant differences in enamel structure loss compared with the non-irradiated group. The group that suffered only erosion and was irradiated with the Er:YAG laser did not had a significant difference from the enamel structure loss values obtained in the non-irradiated group. Following the initial erosion and after treatment (non-irradiated or irradiated with the Er:YAG laser), we observed no significant difference in enamel loss between the groups.

**Figure 3:**
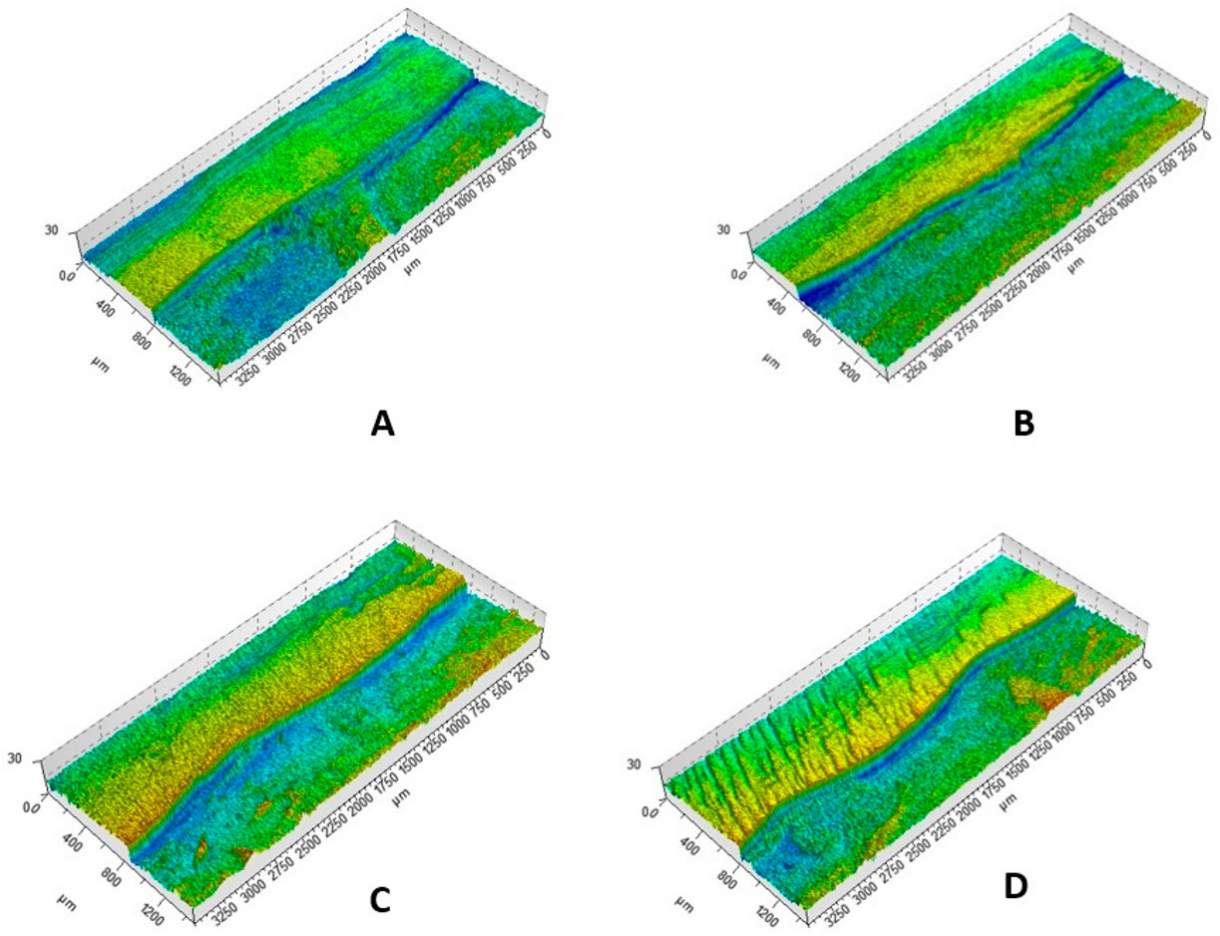
3D optical profilometry images in studied groups. A- Slab irradiated with the Er:YAG laser and eroded during *in situ* phase; B- Slab non-irradiated and eroded during *in situ* phase; C- Slab irradiated with the Er:YAG laser and eroded+abraded during *in situ* phase; D- Slab non-irradiated and eroded+abraded during *in situ* phase

Figure 3 illustrates the 3D images obtained via profilometry analysis, in which the gaps between the sound area (control) and the areas that received erosive challenges, treatment or erosive challenges associated with abrasion can be observed.

### Scanning electron microscopy analysis

**Figure 4:**
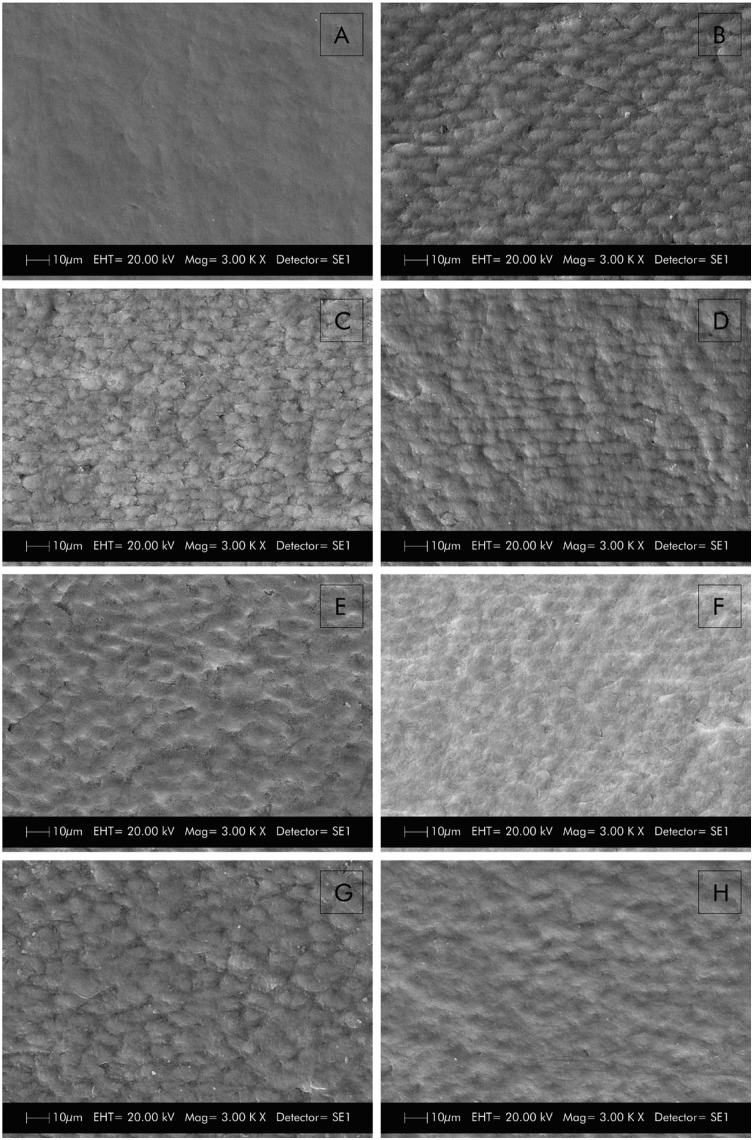
Representative images of scanning electron microscopy analysis. A- Sound surface; B- Initial erosion surface; C- Surface irradiated with the Er:YAG laser; D- Surface non-irradiated; E- Surface irradiated with the Er:YAG laser and eroded during *in situ* phase; F- Surface non-irradiated and eroded during *in situ* phase; G- Surface irradiated with the Er:YAG laser and eroded+abraded during *in situ* phase; H- Surface non-irradiated and eroded+abraded during *in situ* phase

Figure 4A represents a sound enamel surface. Scanning electron microscopy revealed that the immersion in citric acid for 5 min 3 times a day for 2 d promoted the exposure of enamel prisms, which are characteristic of initial erosion lesions (Figure 4B). When this previously eroded substrate received irradiation with the Er:YAG laser, we observed small areas of alteration in the peripheral morphology of the enamel prisms (Figure 4C).

When the specimens were subsequently subjected to the *in situ* erosive challenges, those that had previously been irradiated with the Er:YAG laser (Figure 4E) presented characteristics similar to those of the non-irradiated specimens (Figure 4F), i.e., they exhibited uniform demineralization of the whole surface and dissolution of prisms. Specimens that were subjected to erosive challenges associated with abrasive challenges during the *in situ* phase and that were not treated with the Er:YAG laser (Figure 4H) produced similar images regarding demineralization as specimens that suffered only erosive challenges (irradiated and non-irradiated with the Er:YAG laser). In the specimens subjected to erosion and abrasion and treated with the Er:YAG laser (Figure 4G), it was possible to observe a demineralization pattern of the enamel prisms similar to the area that received only the initial erosion.

## Discussion

Er:YAG lasers (2.94 µm) have been studied regarding the prevention of enamel demineralization and produced positive results when enamel was subjected to cariogenic challenges^4^ ^,^ ^5^. However, few data have shown whether irradiated enamel would react in the same manner when subjected to erosion^12^ ^,^ ^18^ ^,^ ^22^ and erosion associated with abrasion challenges.

Based on the results of this study, our null hypothesis, i.e., that tooth structure loss would be similar in the groups that were irradiated or non-irradiated with an Er:YAG laser and that received erosive challenges and erosive challenges associated with abrasive challenges, was rejected. Irradiation with the Er:YAG laser was not able to control the progression of erosive lesions, as an increase in enamel structure loss was observed following the erosive challenges performed in the intraoral phase. These changes were analyzed via 3D optical profilometry and confirmed through scanning electron microscopy analyses; finding were, in fact, similar to those of a previous study^22^.

When comparing the group that was irradiated with the Er:YAG laser and subjected to abrasion 1 h after the erosive challenge with the group that was irradiated but only subjected to erosion, the first group did not present an increase in enamel structure loss values. However, the group that did not receive irradiation with the Er:YAG laser presented a significant increase in enamel structure loss after being subjected to abrasion when compared with the group that was only subjected to erosion.

Irradiation with an Er:YAG laser may cause morphological changes in the enamel surface^4^, such as an increase in tissue roughness, which may contribute to higher CaF_2_ retention^2^ ^,^ ^29^ from the fluoridated dentifrice, thus leaving the surface more resistant to subsequent erosive challenges. Because of erosion, the accumulation of CaF_2_ on the surface of the enamel can act as a mechanical barrier^25^ and can also disassociate, releasing fluoride ions that combine with hydrogen ions from acidic substances, thus minimizing their potential to promote superficial demineralization^13^. The literature also shows that additional enamel morphological changes, such as the formation of micropores or microcracks caused by laser irradiation, can occur when enamel is irradiated with other types of high power laser (CO_2_ laser), increasing the fluoride uptake into the enamel^6^.

Carvalho, et al.^3^ (2015) observed that erosive challenge with citric acid did not alter the deeper enamel layers (e.g., an erosive challenge performed on the 100 μm layer did not affect the newly ground enamel at a depth of 200 μm). When performing erosive challenges, the enamel layer is superficially demineralized and can be easily removed by brushing. However, as the abrasive process in this study was performed 1 h after the erosive challenges, the use of fluoridated toothpaste may have been able to promote the incorporation of CaF_2_ because this substrate is remineralized by saliva during this waiting time, which prevents an increase in the depth of wear values.

Factors such as film thickness and the time to the maturity of the pellicle may also contribute to the protection of dental enamel against erosion^26^ but may not have the same effect when erosion is associated with an abrasive process. The waiting time before brushing, i.e., 1 h after the erosive challenge, also prevented the softened tooth structure from suffering an immediate effect of tooth-brushing abrasion^19^. Studies have demonstrated that 1 hour is sufficient to ensure enamel protection^27^ because this time allows the saliva to exert its remineralization effect on previously eroded enamel^9^, thereby decreasing the abrasive process^19^.

When evaluating the effect of the Er:YAG laser in association with the erosive challenges, we verified that there was no increase in acid resistance due to irradiation. This result is in agreement with the findings of an *in vitro* study by Reis Dercelli, et al.^18^ (2015), who also identified no protective effect of an Er:YAG laser (60 mJ, 2 Hz, 3.92 J/cm^2^) in the control of enamel wear under erosive challenges with Coca-Cola. The application of subablative parameters can achieve temperatures between 100 and 650°C, which may lead to a reduction of water and carbonate sufficient to alter the crystallinity of the enamel^8^. Deng and Hsu^7^ (2005) observed a reduction of carbonate when enamel specimens were irradiated at energies of 5.1 J/cm^2^, which is similar to the energy levels used in our study (5.2 J/cm^2^). It could be that the cooling that occurred in this study (3 ml/min) caused a reduction of the surface temperature of the tissue, which led only to changes in morphology that contribute to synergy between irradiation with the Er:YAG laser and fluoride toothpaste. This study used parameters that are below the ablation threshold to avoid mechanical damage to the enamel.

In SEM images, we could observe that eroded, non-irradiated specimens had a demineralization pattern with dissolution of the prisms. Following abrasive procedures, we observed a more homogeneous enamel surface, probably due to the removal of the surface layer of the altered prisms, as described by Rios, et al.^20^ (2008), which may have contributed to an increased loss of structure in contrast with the group treated with the Er:YAG laser.

The action of a laser is related to the applied parameters and the type of irradiated substrate, making it difficult to perform direct comparisons with other studies reported in the literature. Future studies in which this structure is chemically evaluated may also contribute to clarifying the mechanisms by which different fluoride compounds associated with fluoride may act to control erosion and abrasion.

## Conclusions

According to the results of this study, although the irradiation with the applied Er:YAG laser did not control the progression of lesions during enamel erosion caused by citric acid, it did control the progression of abrasive lesions in previously eroded enamel.
